# 3-Benzoyl-1-[4-(methyl­sulfan­yl)phen­yl]thio­urea

**DOI:** 10.1107/S1600536813013159

**Published:** 2013-05-18

**Authors:** Rosane de P. Castro, Fernando C. Macedo, Tiago O. Brito, Angelo de Fátima, José R. Sabino

**Affiliations:** a Instituto de Física–UFG, Caixa Postal 131, 74001-970 Goiânia, GO, Brazil; b Departamento de Química–UEL, Caixa Postal 6001, 86051-990 Londrina, PR, Brazil; c Departamento de Química–UFMG, 31270-901 Belo Horizonte, MG, Brazil

## Abstract

The title compound, C_15_H_14_N_2_OS_2_, adopts a helix conformation. An intra­molecular N—H⋯O hydrogen bond leads to a six-membered pseudo-ring [r.m.s. deviation = 0.0212 Å, maximum deviation = 0.033 (1) Å for the N atom bearing the benzoyl group] in the central unit. The benzene and (methyl­sulfan­yl)benzene ring [r.m.s = 0.0028 Å and largest deviation of 0.067 (3) Å for the methyl­sulfanyl C atom] make dihedral angles of 31.76 (8) and 54.68 (6)°, respectively, with the pseudo-ring plane. The dihedral angle between the benzene rings is 85.71 (8)°. In the crystal, pairs of weak N—H⋯S inter­actions form inversion dimers and mediate a linear chain along [001].

## Related literature

For related compounds found in CSD (Allen, 2002 ▶) see: Al-abbasi *et al.* (2010 ▶); Cao *et al.* (1996 ▶). For the structure of the unsubstituted compound, see: Yamin & Yusof (2003 ▶). For details of the synthesis, see: Zhang *et al.* (2001 ▶).
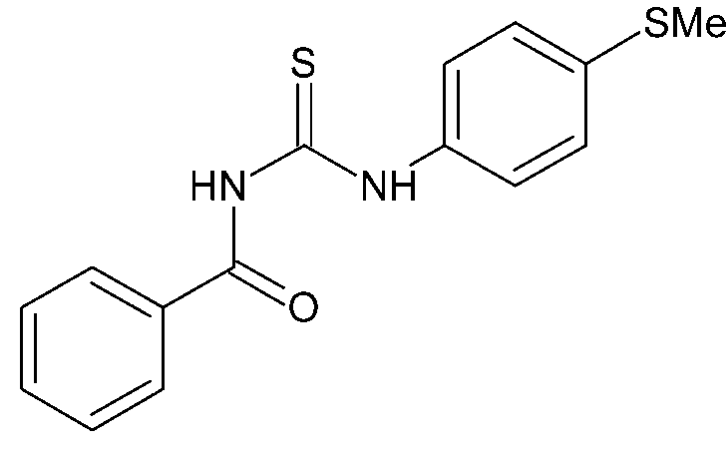


## Experimental

### 

#### Crystal data

C_15_H_14_N_2_OS_2_
*M* *_r_* = 302.42
Triclinic, 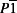

*a* = 5.9131 (2) Å
*b* = 9.5826 (3) Å
*c* = 13.3149 (4) Å
α = 96.729 (1)°
β = 91.533 (1)°
γ = 94.503 (1)°
*V* = 746.46 (4) Å^3^
*Z* = 2
Mo *K*α radiation
μ = 0.35 mm^−1^
*T* = 308 K
0.7 × 0.34 × 0.24 mm

#### Data collection

Bruker APEXII CCD diffractometer
Absorption correction: multi-scan (*SADABS*; Bruker, 2010 ▶) *T*_min_ = 0.917, *T*_max_ = 1.0
11518 measured reflections
3285 independent reflections
2908 reflections with *I* > 2σ(*I*)
*R*_int_ = 0.021

#### Refinement

*R*[*F* ^2^ > 2σ(*F* ^2^)] = 0.034
*wR*(*F* ^2^) = 0.099
*S* = 1
3285 reflections
183 parameters
H-atom parameters constrained
Δρ_max_ = 0.19 e Å^−3^
Δρ_min_ = −0.22 e Å^−3^

### 

Data collection: *APEX2* (Bruker, 2010 ▶); cell refinement: *SAINT* (Bruker, 2010 ▶); data reduction: *SAINT*; program(s) used to solve structure: *SHELXS97* (Sheldrick, 2008 ▶); program(s) used to refine structure: *SHELXL97* (Sheldrick, 2008 ▶); molecular graphics: *ORTEP-3 for Windows* (Farrugia, 2012 ▶); software used to prepare material for publication: *WinGX* (Farrugia, 2012 ▶).

## Supplementary Material

Crystal structure: contains datablock(s) global, I. DOI: 10.1107/S1600536813013159/qm2097sup1.cif

Structure factors: contains datablock(s) I. DOI: 10.1107/S1600536813013159/qm2097Isup2.hkl

Supplementary material file. DOI: 10.1107/S1600536813013159/qm2097Isup3.cml

Additional supplementary materials:  crystallographic information; 3D view; checkCIF report

## Figures and Tables

**Table 1:** Hydrogen-bond geometry (Å, °)

*D*—H⋯*A*	*D*—H	H⋯*A*	*D*⋯*A*	*D*—H⋯*A*
N2—H2⋯O1	0.86	1.93	2.6250 (16)	137
N1—H1⋯S1^i^	0.86	2.61	3.4358 (12)	161

Symmetry code: (i) 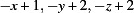.

**Fig. 1.**
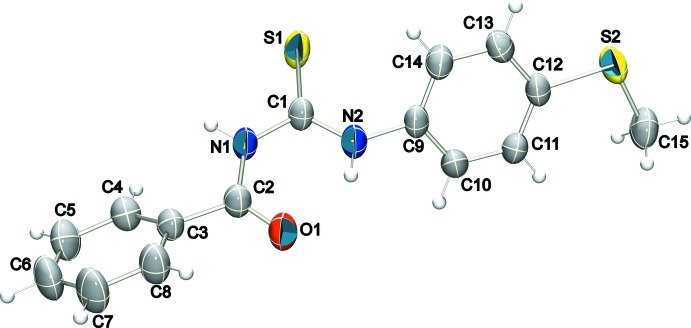
The structure of the title compound showing the atom-numbering scheme. Displacement ellipsoids are drawn at the 50% probability level

**Fig. 2.**
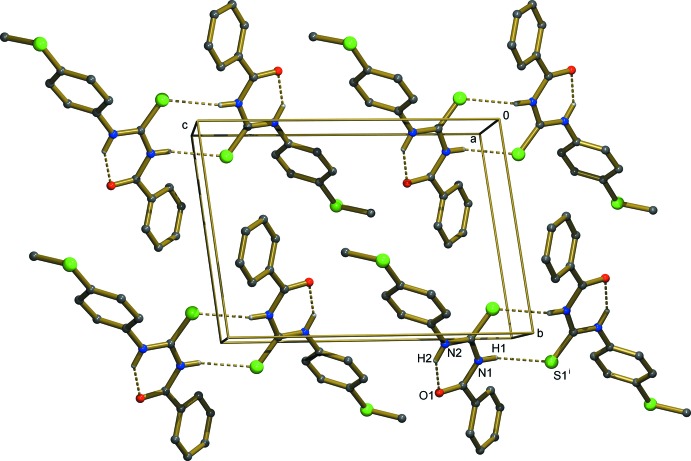
The packing diagram of the title compound. Intermolecular hydrogen bonds are shown as dashed line. Symmetry code: (i) -x + 1, -y + 2, -z + 2.

## supplementary crystallographic information

### Comment

In the title compound, C_15_H_14_N_2_OS_2_, an intramolecular hydrogen bond of type N2—H2···O1 completes a nearly planar six-membered pseudo-ring involving the N2/C1/N1/C2/O1 atoms [r.m.s = 0.0212 Å and largest deviation = 0.033 (1) Å for N1]. The dihedral angle between the benzene ring [r.m.s = 0.0036 Å] and the (methylsulfanyl)benzene ring [r.m.s = 0.0028 Å and largest deviation of 0.067 (3) Å for C15] is 85.71 (8)°. The benzene ring and the (methylsulfanyl)benzene group make dihedral angles of 31.76 (8)° and 54.68 (6)°, respectively, with the plane of the pseudo-ring.

The crystal packing is stabilized by weak N1—H1···S1^i^ [(^i^): -*x* + 1, -*y* + 2, -*z* + 2] interactions (Table 2), which lead to centrosymmetric dimer formation around the inversion center on (1/2,0,0) and arranged in a linear chain along [001]. The helix conformation of the title compound is justified by the supramolecular array.

The bond lengths and bond angles are in agreement with similar benzoylthiourea derivatives found in the CSD (Allen, 2002): 1-Benzoyl-3-(4-hydroxyphenyl)thiourea [CSD refcode:WADSAX (Al-abbasi *et al.*, 2010)], 1-Benzoyl-3-(4-methoxyphenyl)thiourea [CSD refcode: WIRZAY (Cao *et al.*, 1996)] and *N*-Benzoyl-*N*'-phenylthiourea [CSD refcode: HURYAU (Yamin *et al.*, 2003)].

### Experimental

The procedure employed for synthesis of the title compound was described by Zhang *et al.* (2001). Benzoyl chloride (11 mmol) was added to a solution of ammonium thiocyanate (11 mmol) in anhydrous acetone (25 ml). The reaction mixture was heated under reflux for 15 minutes and then cooled to room temperature. A solution of 4-methylthiophenylamine (11 mmol) in acetone (10 ml) was added and the resulting mixture was stirred under reflux for 30 minutes. The reaction mixture was then poured into crushed ice under stirred. The solid product was filtered under and washed with deionized water and purified by recrystallization from ethanol to give fine crystals of the title compound, with an overall yield of 84%.

Spectroscopic data: ^1^H NMR (400 MHz, CDCl_3_, p.p.m.): 2.48 (s, 3H, OCH_3_), 7.27 (dt, J= 8,7 Hz &2,2 Hz, 2H), 7.52 (m, 2H), 7.63 (m, 3H), 7.87 (m, 2H), 9.08 (s, 1H, CONH), 12.54 (s, 1H, CSNH). ^13^C and DEPT-135 NMR (400 MHz, CDCl_3_, p.p.m.): 16.13(+) (OCH_3_); 124.87(+); 127.04(+); 127.68(+); 129.44(+); 131.77; 133.99(+); 134.92; 137.42; 167.14 (*C*=O); 178.34 (*C*=S). FT—IR (KBr, cm^-1^): 3216 ν(amide N—H), 3019 ν(thiourea N—H), 1670 ν(C=O), 1265 ν(C=S).

### Refinement

All H atoms were placed in calculated positions (C–H = 0.93 and 0.96 Å, N–H = 0.86) and treated as riding atoms [*U*_iso_(H) = 1.2*U*_eq_(C,*N*) or*U*_iso_(H) = 1.5*U*_eq_(methyl)]

### Crystal data

**Table:**

C_15_H_14_N_2_OS_2_	*Z* = 2
*M**_r_* = 302.42	*F*(000) = 316
Triclinic, *P*1	*D*_x_ = 1.345 Mg m^−^^3^
Hall symbol: -P 1	Mo *K*α radiation, λ = 0.71073 Å
*a* = 5.9131 (2) Å	Cell parameters from 7480 reflections
*b* = 9.5826 (3) Å	θ = 5.0–54.1°
*c* = 13.3149 (4) Å	µ = 0.35 mm^−^^1^
α = 96.729 (1)°	*T* = 308 K
β = 91.533 (1)°	Prism, colourless
γ = 94.503 (1)°	0.7 × 0.34 × 0.24 mm
*V* = 746.46 (4) Å^3^	

### Data collection

**Table:**

Bruker APEXII CCD diffractometer	2908 reflections with *I* > 2σ(*I*)
Multilayer optics monochromator	*R*_int_ = 0.021
φ and ω scans	θ_max_ = 27.1°, θ_min_ = 1.5°
Absorption correction: multi-scan (*SADABS*; Bruker, 2010)	*h* = −7→7
*T*_min_ = 0.917, *T*_max_ = 1.0	*k* = −12→11
11518 measured reflections	*l* = −17→17
3285 independent reflections	

### Refinement

**Table:**

Refinement on *F*^2^	Secondary atom site location: difference Fourier map
Least-squares matrix: full	Hydrogen site location: inferred from neighbouring sites
*R*[*F*^2^ > 2σ(*F*^2^)] = 0.034	H-atom parameters constrained
*wR*(*F*^2^) = 0.099	*w* = 1/[σ^2^(*F*_o_^2^) + (0.0481*P*)^2^ + 0.2312*P*] where *P* = (*F*_o_^2^ + 2*F*_c_^2^)/3
*S* = 1	(Δ/σ)_max_ = 0.029
3285 reflections	Δρ_max_ = 0.19 e Å^−^^3^
183 parameters	Δρ_min_ = −0.22 e Å^−^^3^
0 restraints	Extinction correction: *SHELXL97* (Sheldrick, 2008)
Primary atom site location: structure-invariant direct methods	Extinction coefficient: 0.038 (4)

### Special details

**Table:**

Geometry. All s.u.'s (except the s.u. in the dihedral angle between two l.s. planes) are estimated using the full covariance matrix. The cell s.u.'s are taken into account individually in the estimation of s.u.'s in distances, angles and torsion angles; correlations between s.u.'s in cell parameters are only used when they are defined by crystal symmetry. An approximate (isotropic) treatment of cell s.u.'s is used for estimating s.u.'s involving l.s. planes.
Refinement. Refinement of *F*^2^ against ALL reflections. The weighted *R*-factor *wR* and goodness of fit *S* are based on *F*^2^, conventional *R*-factors *R* are based on *F*, with *F* set to zero for negative *F*^2^. The threshold expression of *F*^2^ > 2σ(*F*^2^) is used only for calculating *R*-factors(gt) *etc*. and is not relevant to the choice of reflections for refinement. *R*-factors based on *F*^2^ are statistically about twice as large as those based on *F*, and *R*- factors based on ALL data will be even larger.

### Fractional atomic coordinates and isotropic or equivalent isotropic displacement parameters (Å^2^)

**Table:**

	*x*	*y*	*z*	*U*_iso_*/*U*_eq_	
N2	0.6544 (2)	0.96378 (13)	0.72447 (9)	0.0477 (3)	
H2	0.6015	0.8875	0.6881	0.057*	
S1	0.66478 (9)	1.12397 (4)	0.90198 (3)	0.06291 (17)	
C7	−0.0806 (4)	0.4643 (2)	0.83445 (17)	0.0745 (6)	
H7	−0.1007	0.368	0.8133	0.089*	
C4	−0.0192 (3)	0.75005 (17)	0.89870 (12)	0.0506 (3)	
H4	0.0007	0.8462	0.9205	0.061*	
C11	0.9002 (3)	1.18452 (18)	0.53854 (11)	0.0519 (4)	
H11	0.8552	1.2143	0.4776	0.062*	
C1	0.5795 (2)	0.98624 (14)	0.81784 (10)	0.0418 (3)	
C2	0.3113 (3)	0.77171 (15)	0.78408 (11)	0.0460 (3)	
C5	−0.1955 (3)	0.6678 (2)	0.93446 (15)	0.0647 (5)	
H5	−0.2941	0.7087	0.9803	0.078*	
C12	1.1161 (2)	1.22405 (16)	0.57927 (11)	0.0453 (3)	
C13	1.1798 (3)	1.17840 (18)	0.67070 (12)	0.0518 (4)	
H13	1.3244	1.2054	0.699	0.062*	
N1	0.4143 (2)	0.88724 (12)	0.84430 (9)	0.0433 (3)	
H1	0.3711	0.8995	0.9056	0.052*	
O1	0.3658 (2)	0.73896 (13)	0.69735 (9)	0.0679 (4)	
C10	0.7507 (3)	1.10043 (17)	0.58862 (11)	0.0509 (4)	
H10	0.6051	1.0746	0.5611	0.061*	
C3	0.1268 (2)	0.68897 (15)	0.83058 (11)	0.0445 (3)	
C14	1.0303 (3)	1.09336 (18)	0.71986 (12)	0.0504 (4)	
H14	1.0751	1.0624	0.7804	0.061*	
C9	0.8147 (2)	1.05463 (14)	0.67869 (10)	0.0428 (3)	
S2	1.32148 (7)	1.32969 (6)	0.52237 (4)	0.06877 (17)	
C8	0.0945 (3)	0.54539 (17)	0.79760 (14)	0.0604 (4)	
H8	0.1906	0.5041	0.7508	0.072*	
C6	−0.2246 (3)	0.5255 (2)	0.90207 (17)	0.0748 (6)	
H6	−0.343	0.4706	0.9263	0.09*	
C15	1.1792 (3)	1.3752 (2)	0.41292 (15)	0.0684 (5)	
H15A	1.1327	1.2911	0.3684	0.103*	
H15B	1.2801	1.4357	0.3787	0.103*	
H15C	1.0482	1.4235	0.4326	0.103*	

### Atomic displacement parameters (Å^2^)

**Table:**

	*U*^11^	*U*^22^	*U*^33^	*U*^12^	*U*^13^	*U*^23^
N2	0.0555 (7)	0.0429 (6)	0.0431 (6)	−0.0071 (5)	0.0160 (5)	0.0024 (5)
S1	0.0905 (3)	0.0470 (2)	0.0462 (2)	−0.0230 (2)	0.0246 (2)	−0.00146 (16)
C7	0.0898 (14)	0.0473 (9)	0.0854 (14)	−0.0157 (9)	0.0241 (11)	0.0132 (9)
C4	0.0487 (8)	0.0492 (8)	0.0547 (9)	0.0025 (6)	0.0078 (6)	0.0100 (7)
C11	0.0503 (8)	0.0663 (10)	0.0393 (7)	−0.0055 (7)	0.0054 (6)	0.0138 (7)
C1	0.0477 (7)	0.0374 (7)	0.0416 (7)	0.0016 (5)	0.0110 (6)	0.0095 (5)
C2	0.0521 (8)	0.0418 (7)	0.0440 (7)	−0.0021 (6)	0.0100 (6)	0.0065 (6)
C5	0.0528 (9)	0.0740 (11)	0.0698 (11)	0.0033 (8)	0.0211 (8)	0.0161 (9)
C12	0.0423 (7)	0.0493 (8)	0.0451 (7)	0.0007 (6)	0.0133 (6)	0.0085 (6)
C13	0.0373 (7)	0.0671 (10)	0.0525 (8)	0.0004 (6)	0.0061 (6)	0.0148 (7)
N1	0.0512 (7)	0.0402 (6)	0.0384 (6)	−0.0042 (5)	0.0125 (5)	0.0070 (5)
O1	0.0822 (9)	0.0649 (7)	0.0496 (7)	−0.0246 (6)	0.0248 (6)	−0.0069 (5)
C10	0.0470 (8)	0.0623 (9)	0.0406 (7)	−0.0116 (7)	0.0048 (6)	0.0043 (6)
C3	0.0480 (8)	0.0426 (7)	0.0433 (7)	−0.0032 (6)	0.0057 (6)	0.0107 (6)
C14	0.0465 (8)	0.0622 (9)	0.0463 (8)	0.0070 (7)	0.0092 (6)	0.0187 (7)
C9	0.0471 (7)	0.0409 (7)	0.0403 (7)	0.0000 (6)	0.0151 (6)	0.0041 (5)
S2	0.0501 (3)	0.0899 (4)	0.0694 (3)	−0.0149 (2)	0.0089 (2)	0.0348 (3)
C8	0.0725 (11)	0.0450 (8)	0.0630 (10)	−0.0055 (7)	0.0204 (8)	0.0063 (7)
C6	0.0698 (12)	0.0687 (12)	0.0873 (14)	−0.0163 (9)	0.0230 (10)	0.0250 (10)
C15	0.0701 (11)	0.0769 (12)	0.0640 (11)	0.0050 (9)	0.0201 (9)	0.0291 (9)

### Geometric parameters (Å, º)

**Table:**

N2—C1	1.3303 (18)	C5—H5	0.93
N2—C9	1.4331 (17)	C12—C13	1.393 (2)
N2—H2	0.86	C12—S2	1.7635 (14)
S1—C1	1.6643 (15)	C13—C14	1.384 (2)
C7—C6	1.370 (3)	C13—H13	0.93
C7—C8	1.380 (2)	N1—H1	0.86
C7—H7	0.93	C10—C9	1.378 (2)
C4—C3	1.381 (2)	C10—H10	0.93
C4—C5	1.386 (2)	C3—C8	1.391 (2)
C4—H4	0.93	C14—C9	1.380 (2)
C11—C12	1.380 (2)	C14—H14	0.93
C11—C10	1.386 (2)	S2—C15	1.778 (2)
C11—H11	0.93	C8—H8	0.93
C1—N1	1.3890 (17)	C6—H6	0.93
C2—O1	1.2193 (17)	C15—H15A	0.96
C2—N1	1.3783 (18)	C15—H15B	0.96
C2—C3	1.4891 (19)	C15—H15C	0.96
C5—C6	1.377 (3)		
			
C1—N2—C9	126.20 (12)	C2—N1—H1	116.1
C1—N2—H2	116.9	C1—N1—H1	116.1
C9—N2—H2	116.9	C9—C10—C11	120.90 (14)
C6—C7—C8	120.07 (17)	C9—C10—H10	119.5
C6—C7—H7	120	C11—C10—H10	119.5
C8—C7—H7	120	C4—C3—C8	119.70 (14)
C3—C4—C5	119.86 (15)	C4—C3—C2	122.95 (13)
C3—C4—H4	120.1	C8—C3—C2	117.22 (13)
C5—C4—H4	120.1	C9—C14—C13	119.77 (14)
C12—C11—C10	119.89 (14)	C9—C14—H14	120.1
C12—C11—H11	120.1	C13—C14—H14	120.1
C10—C11—H11	120.1	C10—C9—C14	119.61 (13)
N2—C1—N1	116.19 (12)	C10—C9—N2	117.94 (13)
N2—C1—S1	124.91 (11)	C14—C9—N2	122.42 (13)
N1—C1—S1	118.88 (10)	C12—S2—C15	104.62 (8)
O1—C2—N1	122.34 (13)	C7—C8—C3	119.92 (16)
O1—C2—C3	121.47 (13)	C7—C8—H8	120
N1—C2—C3	116.18 (12)	C3—C8—H8	120
C6—C5—C4	119.95 (16)	C7—C6—C5	120.50 (16)
C6—C5—H5	120	C7—C6—H6	119.8
C4—C5—H5	120	C5—C6—H6	119.8
C11—C12—C13	119.09 (13)	S2—C15—H15A	109.5
C11—C12—S2	124.05 (12)	S2—C15—H15B	109.5
C13—C12—S2	116.87 (11)	H15A—C15—H15B	109.5
C14—C13—C12	120.74 (14)	S2—C15—H15C	109.5
C14—C13—H13	119.6	H15A—C15—H15C	109.5
C12—C13—H13	119.6	H15B—C15—H15C	109.5
C2—N1—C1	127.82 (12)		
			
C9—N2—C1—N1	176.58 (13)	O1—C2—C3—C8	30.6 (2)
C9—N2—C1—S1	−2.0 (2)	N1—C2—C3—C8	−150.00 (15)
C3—C4—C5—C6	0.0 (3)	C12—C13—C14—C9	−0.8 (2)
C10—C11—C12—C13	−0.1 (2)	C11—C10—C9—C14	0.2 (2)
C10—C11—C12—S2	179.62 (12)	C11—C10—C9—N2	−177.77 (14)
C11—C12—C13—C14	0.7 (2)	C13—C14—C9—C10	0.4 (2)
S2—C12—C13—C14	−179.05 (12)	C13—C14—C9—N2	178.27 (13)
O1—C2—N1—C1	3.7 (3)	C1—N2—C9—C10	−124.24 (17)
C3—C2—N1—C1	−175.71 (13)	C1—N2—C9—C14	57.8 (2)
N2—C1—N1—C2	−3.1 (2)	C11—C12—S2—C15	3.27 (17)
S1—C1—N1—C2	175.56 (12)	C13—C12—S2—C15	−177.03 (13)
C12—C11—C10—C9	−0.4 (2)	C6—C7—C8—C3	1.1 (3)
C5—C4—C3—C8	0.6 (2)	C4—C3—C8—C7	−1.2 (3)
C5—C4—C3—C2	176.20 (15)	C2—C3—C8—C7	−177.03 (17)
O1—C2—C3—C4	−145.13 (17)	C8—C7—C6—C5	−0.5 (4)
N1—C2—C3—C4	34.3 (2)	C4—C5—C6—C7	−0.1 (3)

### Hydrogen-bond geometry (Å, º)

**Table:**

*D*—H···*A*	*D*—H	H···*A*	*D*···*A*	*D*—H···*A*
N2—H2···O1	0.86	1.93	2.6250 (16)	137
N1—H1···S1^i^	0.86	2.61	3.4358 (12)	161

Symmetry code: (i) −*x*+1, −*y*+2, −*z*+2.
